# My favourite flowering image: a capitulum of Asteraceae

**DOI:** 10.1093/jxb/erw489

**Published:** 2017-01-30

**Authors:** Paula Elomaa

**Affiliations:** 1 Department of Agricultural Sciences, Viikki Plant Science Centre, University of Helsinki, Finland; 2 Swedish University of Agricultural Sciences

**Keywords:** Asteraceae, capitulum, gerbera, inflorescence, phyllotaxis, sunflower

**I could have selected my favourite flowering image from thousands of astonishing images that have captured the geometric regularity of head-like inflorescences in Asteraceae. These unique inflorescences pack hundreds of flowers into precise spirals whose numbers follow a famous mathematical rule. Meanwhile, the whole structure may mislead a layman (or a pollinator) by mimicking a single solitary flower although it consists of morphologically and functionally distinct types of flowers.**

**Fig. 1.**
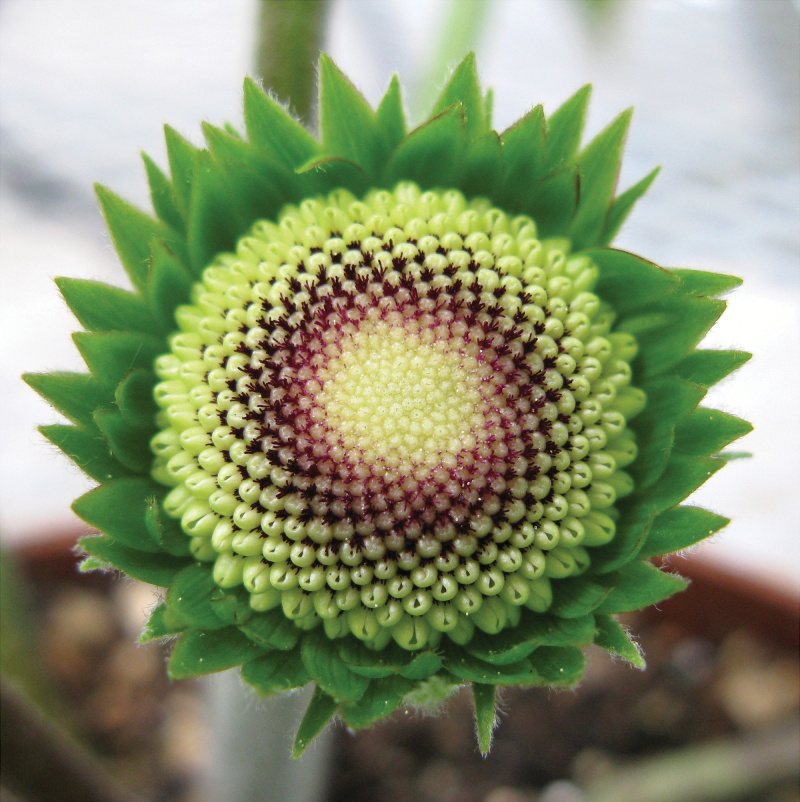
A composite inflorescence of *Gerbera hybrida* showing spiral phyllotaxis of emerging flowers. Hundreds of flowers are packed into the capitulum that mimics a solitary flower and is surrounded by green, involucral bracts.

Regular, reproducible patterns in nature are fascinating and inspiring. They represent enigmatic mathematical and biological problems but also provide inspiration and aesthetic delight that has impacted, for example, arts and architecture. Inflorescence architecture in terms of arrangement of flowers in branched systems provides an example of this geometric regularity that is not only fascinating *per se* but affects crop yield, fitness, and adaptation of plants. The flowering image I selected represents a classical example of spiral phyllotaxis found in composite inflorescences such as sunflower (*Helianthus* sp.). This image (Fig. 1), however, is of a commercial cultivar of the ornamental crop *Gerbera hybrida*, the model species that our lab has worked on for more than 25 years. This structure keeps on bringing surprises and moments of joy year by year.

The composite inflorescence, capitulum, or flower head in Asteraceae assembles multiple flowers into a single, highly compressed structure. It is a very effective reproductive unit and with an apparent selective value, considered to be the key innovation for diversification of this largest family of flowering plants. In the case of sunflower or gerbera, hundreds of individual flowers are all attached to a flat enlarged receptacle. As beautifully visible in the image, the developing flowers are organized into left- and right-winding spirals (parastichies). Intriguingly, the number of these spirals follow two consecutive numbers of Fibonacci series (1, 1, 2, 3, 5, 8, 13, 21, 34 … where each number is the sum of the two preceding numbers in the series). In the particular cultivar in my favourite image, there are 34 clockwise and 21 anti-clockwise spirals (you may check this!) while our standard model cultivar, Terra Regina, typically shows 55 and 34 spirals, respectively. Interestingly, although the Fibonacci numbers clearly dominate, non-Fibonacci structures such as double Fibonacci numbers (2, 4, 6, 10, 16, 26…), Fibonacci ±1 or Lucas numbers (1, 3, 4, 7, 11, 18…) do appear, as recently demonstrated in a large citizen science experiment engaging the public to grow and count sunflower spirals (Swinton *et al.*, 2016). It seems that there are always exceptions to the rule; the experiment revealed cases where spirals were countable but not Fibonacci, as well as well-defined parastichies in only one direction or even uncountable structures. In a shoot apex, the spiral emergence of leaf primordia follows an approximate golden angle (137.5°), and their positioning in the fist available space between already existing primordia is associated with formation of auxin maxima at incipient primordia and on subsequent depletion of auxin in their vicinity (Reinhardt *et al.*, 2003; Heisler *et al.*, 2005). However, it is still unclear what are the developmental mechanisms that regulate the organization of the expanded capitulum that is much larger in its dimensions, and how they are linked to observed deviations of the Fibonacci structure. Mathematical modelling can reproduce many of these remarkable patterns (e.g. Douady and Couder, 1996; Smith *et al.*, 2006; Zagórska-Marek and Szpak, 2008; Owens *et al.*, 2016), and have already provided inspiring hypotheses to be tested at the molecular level.

The visual attractiveness of composite inflorescences is further enhanced by the presence of distinct flower types. Normally, gerbera (like sunflower) has showy, large and bilaterally symmetrical marginal ray flowers, and smaller, more radially symmetrical central disc flowers. The image I selected is taken from a cultivar that only develops male sterile ray-like flowers (so-called crested phenotype; Kloos *et al.* 2004; Broholm *et al.*, 2014 ; Juntheikki-Palovaara *et al.*, 2014). The image represents an early developmental stage of the inflorescence and, therefore, the showy petals have not yet reached their full size. The famous painting by Vincent van Gogh with double-flowered sunflower heads has preserved a similar mutant phenotype in the history of art (Chapman *et al.*, 2012). The molecular studies in gerbera (Broholm *et al.*, 2008), *Senecio* (Kim *et al.*, 2008), as well as sunflower (Chapman *et al.*, 2012) all indicated that CYCLOIDEA-like TCP domain transcription factors, among the key developmental regulators in plants, have been recruited to regulate capitulum architecture. This gene family has expanded in Asteraceae and consequently evolved novel functions in regulating ray identity. In the crested gerbera as well as in double-flowered sunflower, up-regulation of a CYC-like TCP gene converted the disc flowers into ray-like flowers by affecting the growth of the petals, and disrupting stamen development (Chapman *et al.*, 2012; Juntheikki-Palovaara *et al.*, 2014).

The Asteraceae inflorescences are false flowers (pseudanthia) that mimic solitary flowers. As visible from the image, the capitulum is surrounded by involucral bracts (the bright green leaf-like organs) that perform a sepal-like, protective function. The showy ray flowers can be seen to be analogous to attractive petals in solitary flowers, and the hermaphrodite disc flowers to reproductive organs (carpels/stamens). By conducting functional analyses for the gerbera orthologues of flower meristem identity genes *LEAFY* (*LFY*) and *UNUSUAL FLORAL ORGANS* (*UFO*), we recently discovered that the capitulum resembles a solitary flower also at the molecular level (Zhao *et al.*, 2016). As in single flower meristems in Arabidopsis, the gerbera *GhLFY* expression was found to be uniform across the naked inflorescence meristem, defining it as a determinate structure. Loss of *GhLFY* expression led to loss of determinacy of the capitulum and disrupted phyllotaxis. In contrast, by ectopic expression of *GhUFO*, this large structure gained floral fate; instead of flower primordia, the meristem initiated numerous flower organ primordia arranged in whorled phyllotaxis (Zhao *et al.*, 2016). Our studies also provided the first molecular evidence to explain the evolutionary origin of flower types. Several botanical studies have indicated that although ray flowers are located along the Fibonacci spirals, they show delayed development compared with adjacent disc (or trans) flowers (Harris, 1995; Bello *et al.*, 2013). In an extreme case, ray flowers initiate after the disc flowers and their development proceeds in a different direction, namely towards the margins of the head (Harris *et al.*, 1991). We showed that GhLFY has evolved a novel function in regulating the early ontogeny of ray flowers in gerbera (Zhao *et al.*, 2016). Silencing of *GhLFY* converted ray flowers into branched structures resembling those found in Calyceraceae, the phylogenetically closest relatives of Asteraceae (Pozner *et al.*, 2012). Our data thus indicated that, during evolution, GhLFY has played a major role in contributing to the gain of floral fate for these peripheral branches still found in capitula of Calyceraceae, and that the differential development of ray flowers relates to their different ontogenic origin from separate branching systems.

Although some details on the gene functions and molecular networks controlling capitulum architecture and flower type differentiation have been revealed, the future challenge is to understand the dynamics of early patterning of the inflorescence meristem and establishment of the spiral phyllotaxis. Classical experiments in sunflower already showed that patterning can be disrupted by wounding (Palmer and Marc, 1982; Hernandez and Palmer, 1988, 1990). Interestingly, wounding creates a new margin that resets patterning and initiates successive formation of new bracts, rays, and discs, in this particular order. This raises a still unresolved fundamental question; how does the margin specify initiation of organ/flower primordia and what is the nature of the signal that it creates? Furthermore, an extra level of complexity in species developing capitula within capitula (syncephalium) surely adds to the number of variations on a theme.
